# Supplementary material for: Repurposing Disulfiram (Tetraethylthiuram Disulfide) as a Potential Drug Candidate against Borrelia burgdorferi In Vitro and In Vivo
Source: Antibiotics (Basel). 2020 Sep 22;9(9):633. doi: 10.3390/antibiotics9090633 (PMC7557442; doi:10.3390/antibiotics9090633)
Supplement: Supplementary file 1 [file antibiotics-09-00633-s001.pdf]

Supplemental Figures

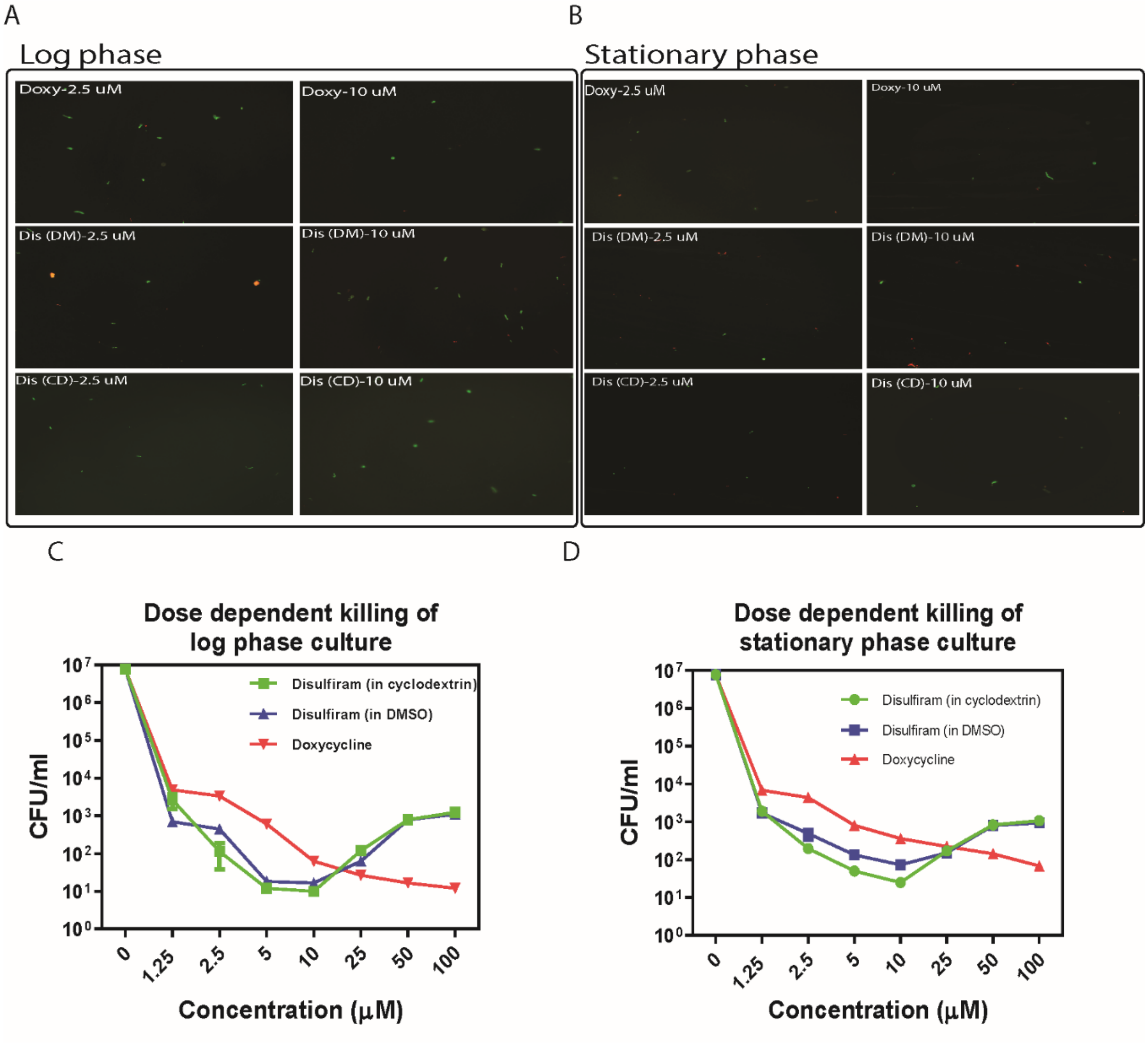

Figure S1. Representative images were taken for both Log phase (**A**) and Stationary phase (**B**) using SYBR green-fluorescent stain (live organisms) and Propidium Iodide red-fluorescent stain (dead organisms) at 200× magnification. Dose dependent killing of B. burgdorferi by antibiotics. (**C**) A log phase culture and (**D**) stationary phase culture. The exponential culture of B. burgdorferi culture was exposed to antibiotics, and surviving cells were determined by CFU count. The culture was treated with disulfiram (in cyclodextrin), disulfiram (in DMSO), and doxycycline (*n* = 3). Error bars represent standard errors.

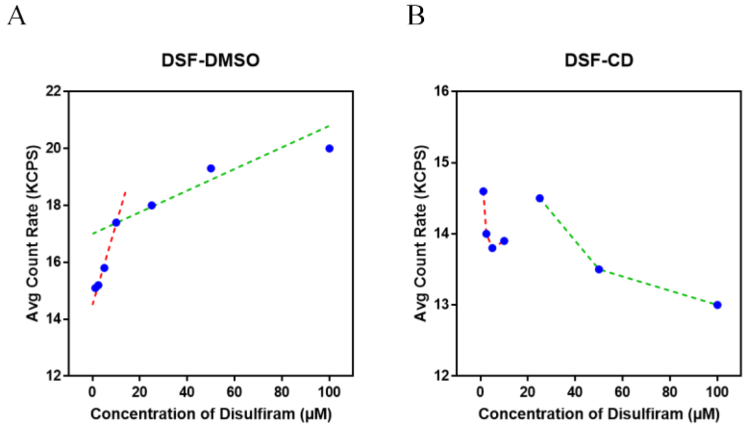

Figure S2. Dynamic light scattering (DLS) analysis of disulfiram at different concentration in presence of 5% (v/v) bovine serum albumin (BSA) solution. The disulfiram solution was prepared by diluting the concentrated disulfiram stock solutions dissolved in either in **(A)** DMSO or in **(B)** hydroxypropyl β-cyclodextrin (30% w/v) solution. The average count rate at different concentrations of disulfiram indicate two different aggregation patterns with a critical concentration at 10 μM. The red and green dotted lines are trend lines that represent below and above the critical aggregate concentration (CAC) respectively.

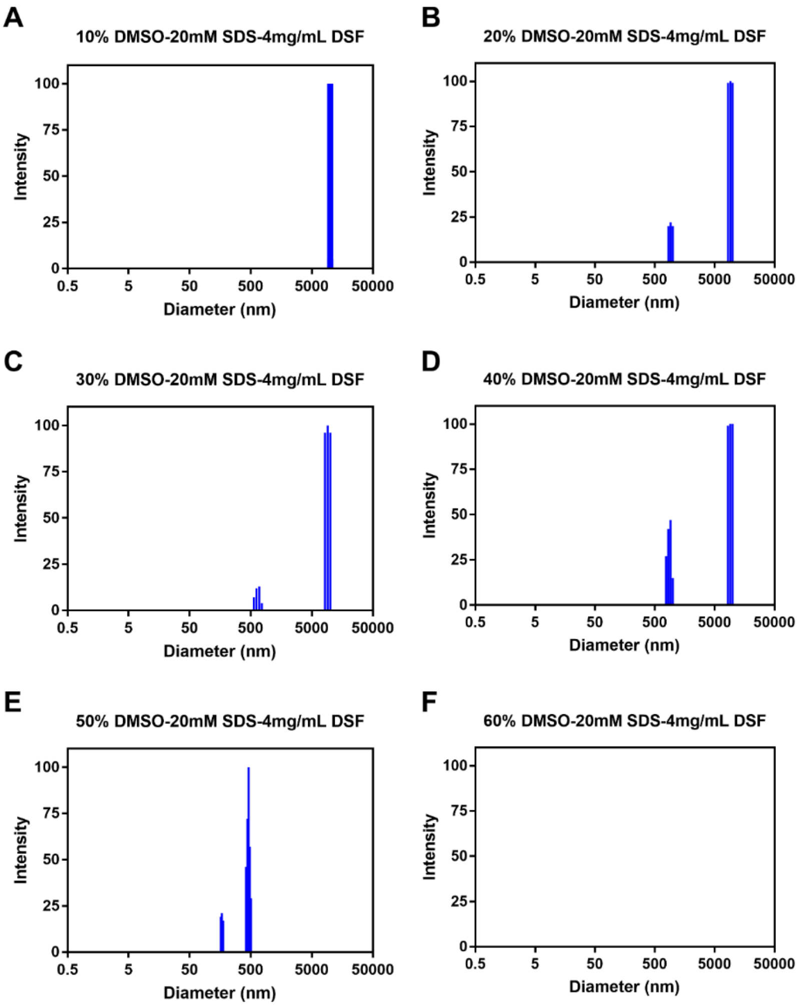

Figure S3. DLS analysis of disruption of disulfiram aggregation study, Panels A-F are showing increasing percentage of DMSO in the presence of 20 mM SDS and disulfiram (DSF) concentration of 4 mg/ml (high concentration, well-above critical aggregate concentration (CAC)). The DSF larger aggregates of approx. 10μ exists up to 40 % DMSO, and DSF disaggregates, and all the particles disappear at 60% DMSO and above.

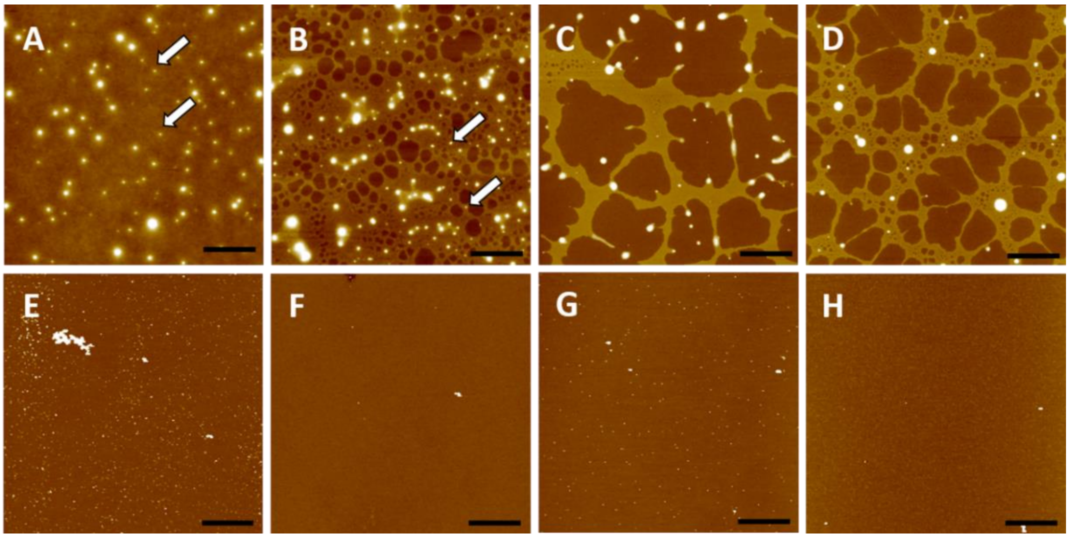

Figure S4. AFM images of disulfiram formulations prepared in cyclodextran **(A–D)** and DMSO **(E–H)** in the following solution concentrations: A,E–100 μM, B,F–25 μM, C,G–10 μM, D,H–5 μM. White arrows mark small size aggregates in the images which formed regardless of local aggregation due to solution drying during sample preparation. Vertical scale contrast is same for all images (4 nm). Scale bar is 2 μm.

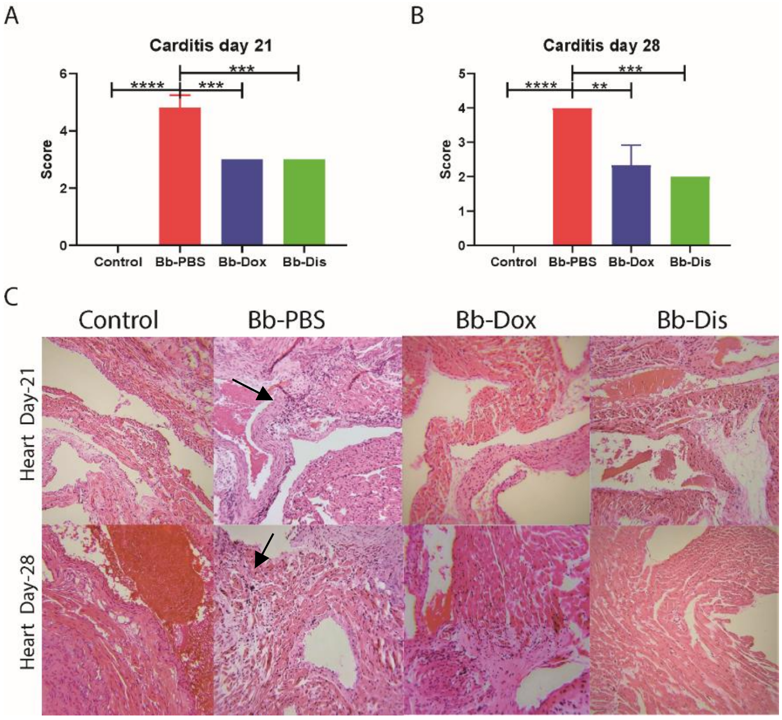

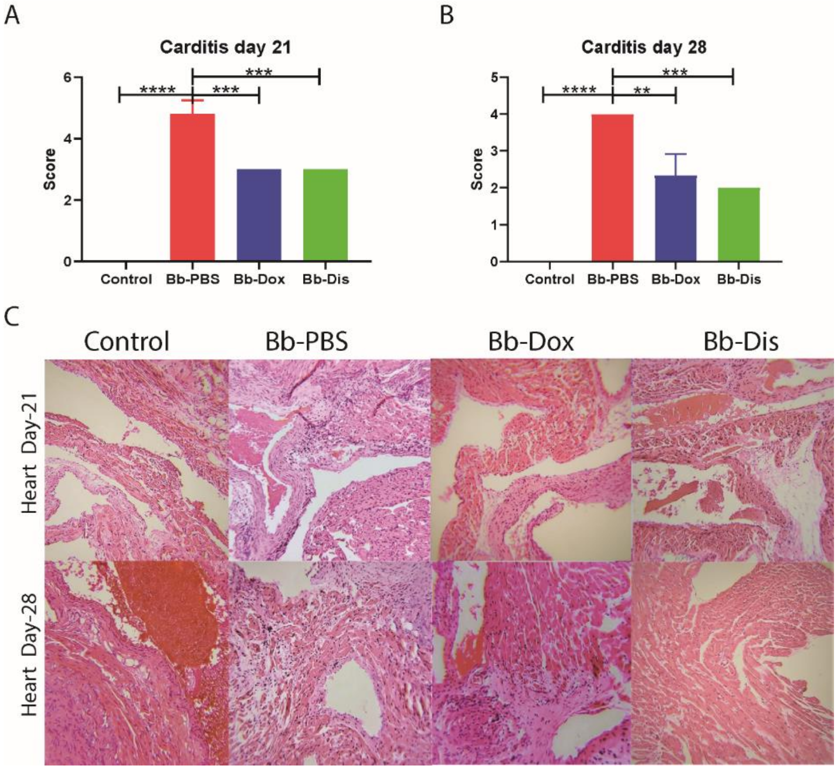

Figure S5. A & B. Histopathology was empirically quantified by scoring carditis blindly in 5 fields per sample and averaging per group. **C.** Photomicrographs (10×) of hematoxylin and eosin stained heart sections; arrows depict the mono nuclear leucocyte infiltrates. Statistics by unpaired t test with Welch's correction between controls versus infected and also between drugs treated group versus infected group. ** $p < 0.01$, *** $p < 0.001$, ****$p < 0.0001$.

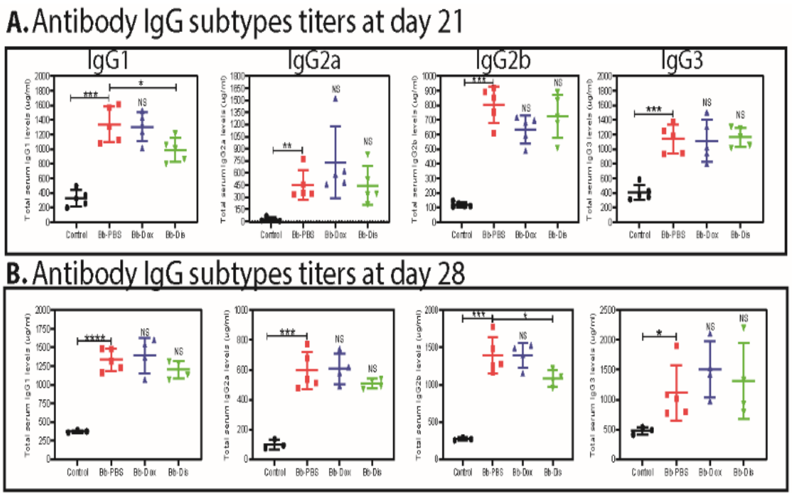

Figure S6. Antibody response in the serum with and without antibiotics treatment which were quantified by ELISA. Total concentration of IgG1, IgG2a, IgG2b and IgG3 antibodies **A**. day 21 post-infection and **B**. day 28 post-infection. Statistics: unpaired t test with Welch's correction between controls versus infected and between drug-treated group versus infected group. *$p < 0.05$, ** $p < 0.01$, *** $p < 0.001$. NS means not significant.
